# Supplementary material for: The social implications of participant choice on adherence to Isonaizid Preventive Therapy (IPT): A follow-up study to high completion rates in Eswatini
Source: PLoS One. 2020 May 29;15(5):e0232841. doi: 10.1371/journal.pone.0232841 (PMC7259658; doi:10.1371/journal.pone.0232841)
Supplement: S1 File — (DOCX) [file pone.0232841.s001.docx]

**Identifying Critical Factors to Enable Successful Delivery and Treatment Completion of Isoniazid Preventive Therapy in Swaziland**

**RESEARCH PROJECT INFORMATION**

Hello. My name is ……............ Thank you for agreeing to talk to me about factors that influence the uptake of IPT in Swaziland. I am contacting you because you participated in the 2015 IPT study.

**What language do you prefer for our discussion today?**

Siswati English

This research project is being conducted by researchers from Dartmouth’s Geisel School of Medicine in Hanover, NH, USA, and the University Research Co. in Mbabane, with endorsement from the Swaziland Ministry of Health. It is a study to understand the things that helped people like you, those who were in a TB study last year, to take their medicine every day from start to finish.

Being a part of this research is up to you. You do not have to agree to answer any questions. If you do agree, we will ask you to answer questions for 30 minutes about your experience of taking a medicine for six months to protect you from TB. You can answer all or none of the questions. You may ask to stop the interview at any time. If you decide not to answer any questions, it will not affect the care that you are receiving at the clinic. The information we get from you will be used for future work to help people take their IPT. Your answers will not be shared with anyone outside our research group. If you agree, we may use parts of what you say to us in presentations and papers concerning this project. We will never use your name or other information that might reveal your identity in any presentation, report or paper.

Questions about this project may be directed to:

Lisa Adams, MD

Associate Professor of Medicine, Associate Dean for Global Health,

Dean’s Building, 1 Rope Ferry Road, Geisel School of Medicine at Dartmouth, Hanover NH 03755-3525

Phone: 001.603. 650.6063 Fax: 603.650.1202 Email: [Lisa.V.Adams@dartmouth.edu](mailto:Lisa.V.Adams@dartmouth.edu)

or

Samson M. Haumba, MBChB, M. Med, MPH, DLit et Phil

Country Director, University Research Co., LLC-Swaziland

Mbabane Office Park, Sozisa Road; PO. Box 1404, Mbabane;

Phone: 268 76026400 or 268 2404 7154/6. Email: [SamsonH@urc-sa.com](mailto:SamsonH@urc-sa.com)

***I’m interested in learning about your experiences when you go try to get general health care services.***

| **Besides this clinic, is there anywhere else you go for care when you don’t feel well?** (tick all that respondent says – do not prompt) |
| --- |
| Another government clinic |
| Private clinic |
| Spiritualists |
| Traditional healers |
| Other |
| None |
|  |
| **For what types of problems do you go to the health clinic?** |
|  |
| **What do you think about the quality of health services available in your community?** |
| Very good |
| Good |
| Bad |
| Very bad |
| Comments |
|  |
| **Can you describe how most people in your community think about quality of the health care that is available to them in this clinic?** |
| Very good |
| Good |
| Bad |
| Very bad |
| Comments |
|  |
| **How do you think that the clinic staff interacts (behaves) with patients? (Possible probe: I mean, is the staff respectful/disrespectful, kind/unkind, gentle/rough, patient/impatient, etc?)** |
|  |
|  |
| **What do you think about availability or access to health services in your community?** |
| Very good |
| Good |
| Bad |
| Very bad |
| Comments |
|  |
| **What influenced you to choose the health clinic where you receive ART? [open ended – do not prompt]** |
| Proximity to home/work |
| Privacy/confidentiality |
| Ease of access |
| Referral from family and friends |
| Trust the clinician |
| Comments |
|  |
| **When talking with your clinician about your health/treatment services, what is most important to you?** |
| Friendly |
| Respectful |
| Competent |
| Other |
| Comments |
|  |
| **When you get care at the clinic, what features matter most to you?** |
| Available services |
| Short wait times |
| Other |
| Comments |

*______________________________________________________________________________________*

***Now I’d like to know about your experiences while you were in the IPT study.***

**Please tell me what motivated you to participate in the IPT study.**

Didn’t want to get TB

Pressured by clinician

Thought I’d get special treatment

Comments

**Please tell me why you think it is important for HIV-infected patients to take IPT.**

HIV gets better

Prevents TB

Live longer

Comments

**Did you tell anyone that you were in this study?**

- No, I did not tell anyone I was in the study

If not, why not?

- Yes, I did tell someone I was in the study.

If yes, who did you tell? (Can prompt with below choices.)

- Family
- Friends
- Employer
- Others…………………..……………………………………………………………………………………………………………………..

If yes, why did you tell them?

If yes, do you think that telling them may have helped you complete IPT? Please explain.

**Was anything different about how the clinicians described IPT to you (compared to how they usually describe a medicine not part of a study)?**

- Yes
- No
- I don’t know

Comments

**Can you please describe what could work better in a study like this in the future?**

***When a person takes IPT, they should take as much of their treatment as possible so they get maximal benefit. I want to talk to you about how you managed to complete your IPT.***

**How long did it take you to complete your IPT? ……… months**

**If longer than 6 months, do you remember why it took longer than 6 months?**

**What aspects/factors helped you to take your INH pills every day? (Prompt if necessary: Was there anything that helped you to remember to take your INH pills?)**

**Did you ever miss a pill during the course of your IPT?**

- No
- Yes

If yes, do you remember what the reasons were for that? (Allow for more than one reason)

- I don’t remember

**Think about when you were on IPT in the study.**

**What helped you to complete your IPT?**

**What made it hard for you to complete your IPT?**

**Do you think that being in this research study helped you complete your IPT? Please explain.**

***In this study, people were asked how they wanted to get their INH pills – at the clinic or in their community. I want to talk to you about your choice.***

**Did you like that you had a choice about how to get your INH pills?**

- Yes
- No
- I don’t know

Comments

**Sometimes your clinician just tells you how to take your treatment or medicine without asking for your opinion. Do you like this approach?**

- Yes
- No
- I don’t know

Comments

**Sometimes the patient and clinician make choices about a patient’s treatment together. Do you like this approach?**

- Yes
- No
- I don’t know

Comments

**Did the clinic staff tell you which way to get your IPT? (Prompt if necessary: Did anyone pressure you to choose getting your pills in the clinic or community?)**

- Yes
- No
- I don’t know

Comments

***_***

***________________________________________________________________________***

[**The next part is ONLY for those patients who took any of their IPT in the facility-based model i.e., either started or switched to facility-based IPT. If this patient did not take their IPT in a facility, skip this section.]**

***Our records show you chose to get at least some of your IPT at the clinic. I’m going to ask you questions about that decision now.***

| **How did you travel to the clinic for your IPT? Check all that apply.** |
| --- |
| Walk |
| Bus |
| Kombi |
| Own Car |
| Other specify |
| Comments |
|  |
| **Did anyone come with you on some or most days?** |
| Yes |
| If yes, who? |
| No |

**Please tell me why you chose to get your IPT at the clinic instead of in the community.**

**When did you fit in going to the clinic in your typical day while you were in the study? (Prompt if necessary: For example, did you stop at the clinic on your way to work?)**

**Describe your typical day while you were getting your IPT at the clinic.**

**_______________________________________________________________________________________**

**[This part is ONLY for those who took any of their IPT through the community-based model i.e., either started with or switched to community-based IPT. If this patient did not take their IPT in the community, skip this section.]**

***Our records show you chose to get at least some of your IPT in the community. I’m going to ask you questions about that decision now.***

| **Where did you get your IPT? Check all that apply.** |
| --- |
| Home |
| Work |
| Church |
| Shop |
| Other specify |
| Comments |
|  |
| **Was anyone with you on some or most days?** |
| Yes |
| If yes, who? |
| No |

**Please tell me why you chose to get your IPT in the community instead of at the clinic?**

**Describe your typical day while you were getting your IPT in the community.**

**_______________________________________________________________________________________**

***People in the study were given the chance to change the way that they got their IPT. I want to understand more about that.***

**Did you switch where you received your IPT? For example, did you start getting your pills in your home then switch to getting them at the clinic?**

- Yes

If yes, why did you switch?

- No, I didn’t switch
- I don’t know

**Did you switch the place you received your IPT more than once during the study?**

- Yes

If yes, why did you switch more than once?

- No, I didn’t switch more than once
- I don’t know

**______________________________________________________________________________________**

***Now in the last of our time together, I would like to know what you think about using IPT throughout Swaziland***

**In your opinion, what are the biggest barriers to getting more patients on IPT in Swaziland?**

**What do you think are the biggest barriers for a patient to complete IPT in Swaziland?**

**Do you think it is important to link picking up the IPT pills with picking up the ARVs?**

- Yes
- No
- I don’t know

Comments

**Do you think it helped you to complete IPT because you were offered a choice of how to receive it?**

- Yes
- No
- I don’t know

Comments

**Do you think you were more likely to complete IPT because the nurses/research staff treated you any differently than other patients who weren’t in the study? (Possible prompt: shorter lines, nicer, etc?)**

- Yes
- No
- I don’t know

Comments

***Thank you very much for your time and participation. Here is an envelope with your participation stipend.***
