# Supplementary material for: The social implications of participant choice on adherence to Isonaizid Preventive Therapy (IPT): A follow-up study to high completion rates in Eswatini
Source: PLoS One. 2020 May 29;15(5):e0232841. doi: 10.1371/journal.pone.0232841 (PMC7259658; doi:10.1371/journal.pone.0232841)
Supplement: S2 File — (DOCX) [file pone.0232841.s002.docx]

**Identifying Critical Factors to Enable Successful Delivery and Treatment Completion of Isoniazid Preventive Therapy in Swaziland**

**Kwatiswa Mayelana Nalolucwaningo**

Lolucwaningo lwentiwa bacwaningi base Dartmouth’s Geisel School of Medicine in Hanover, NH, USA, kanye ne University Research Co. e-Mbabane. Lolucwaningo luvunyelwe Litiko Letemphilo eSwatini. Inhloso yalolucwaningo ikutsi sitfole loko lokwanta kutsi bantfu labafana nawe, labebakulolucwaningo lweTB emnyakeni lophelile, bakhona kunatsa baze bawacendza emaphilisi ekuvikela i-TB.

Kuba yincenye yalolucwaningo kukutitsandzela kwakho. Awukaphocelelwa kutsi uphendvule noma ngumuphi umbuto. Nawuvuma kuba yincenye yalolucwaningo, sitakucela kutsi uphendvule imibuto letsite lengatsatsa sikhatsi lesingaba yimizuzu lengemashumi lamane (40 minutes). Lencociswano yetfu sitayitsebula kuze ngingatfole kahle konkhe lesikhulume ngako. Lemibuto itawutsintsa indlela lowativa ngayo lapho uniketwa futsi usebentisa emaphilisi ekuvikela i-TB kuto tonkhe letinyanga letisitfupha lobowunatsa ngato emaphilisi ekukuvikela ku-TB. Ungayiphendvula yonkhe lemibuto noma wale kuphendvula ngisho munye. Ungacela nekuyimisa lencociswano noma nini. Nawukhetsa kungabi yincenye yalolucwaningo, kwelashwa kwakho lapha emtfolamphilo angeke kutsikameteke. Lokwatisa lotasinika kona kutawusebentiselwa kusita labanye esikhatssini lesitako kutsi bakwati kunatsa kahle emaphilisi abo. Timphendvulo takho anageke sitinikete labanye bantfu ngaphandle kwalabo labasebenta kulolucwaningo. Nawuvuma, sitawusebentisa lokunye kwaloku lositjele kona nasitfula imiphumela yalolucwaningo. Angeke silisebentise noma livele ligama lakho noma-ke lokunye kwatisa lokungenta kutsi lomunye umuntfu ati kutsi lokwatisa sikutsetse kuwe noma nini nasitfula imiphumela yalolucwaningo.

Nawunemibuto ngalolucwaningo, ungabuta:

Lisa Adams, MD

Associate Professor of Medicine, Associate Dean for Global Health,

Dean’s Building, 1 Rope Ferry Road, Geisel School of Medicine at Dartmouth, Hanover NH 03755-3525

Phone: 603. 650.6063 Fax: 603.650.1202 Email: [Lisa.V.Adams@dartmouth.edu](mailto:Lisa.V.Adams@dartmouth.edu)

Noma

Samson M. Haumba, MBChB, M. Med, MPH, DLit et Phil

Country Director, University Research Co., LLC-Swaziland

Mbabane Office Park, Sozisa Road; PO. Box 1404, Mbabane; Tel: 268 76026400 or 268 2404 7154/6. [SamsonH@urc-sa.com](mailto:SamsonH@urc-sa.com)

**Identifying Critical Factors to Enable Successful Delivery and Treatment Completion of Isoniazid Preventive Therapy in Swaziland**

**Kwatiswa Mayelana Nalolucwaningo**

Sawubona. Ligama lami ngingu …………………………Ngiyabonga kuvuma kukhulumisana nami ngetinsita letitfutfukisa kusebentiswa kwe-IPT eSwatini.

**Nguluphi lulwimi longatsandza silusebentise namuhla?**

Siswati English

***Read the consent statement and tear off and give that page to the patient.***

**Ngaphambi kwekutsi sicale kucoca, mhlawumbe ungatsandza kutfola i-drink?**

***Ngifise kuva ngendlela lophatseka ngayo nawutfola lusito lwetemphilo***

**Experiences of receiving health care services**

| **Question** |  |  |  |
| --- | --- | --- | --- |
| **Ngaphandle kwalomtfolamphilo lona, kukhona yini lapho uya khona kute utfole lusito lwetemphilo nawutiva uphatsekile/ ugula?** (tick all that respondent says – do not prompt) |  |  |  |
| Another government clinic |  |  |  |
| Private clinic |  |  |  |
| Spiritualists |  |  |  |
| Traditional healers |  |  |  |
| Other |  |  |  |
| No |  |  |  |
| **Uyela tiphi nje tinkinga tetemphilo emtfolamphilo?** |  |  |  |
|  |  |  |  |
|  |  |  |  |
|  |  |  |  |
| **Ucabanga kutsi lunjani lusito lwetemphilo lolukhona emangweni wakho?** |  |  |  |
| Very good |  |  |  |
| Good |  |  |  |
| Bad |  |  |  |
| Very bad |  |  |  |
| Comments: |  |  |  |
| **Ungingichazela kutsi bantfu labanengi emangweni wakini bacanga kutsi lusito lwetemphilo lolutfolakala kulomtfolamphilo?** |  |  |  |
| Very good |  |  |  |
| Good |  |  |  |
| Bad |  |  |  |
| Very bad |  |  |  |
| **Ucabanga kutsi tisebenti temphilo (nurse/dokotela) letikulomtfolamphilo tikhulumisana njani nebantfu labete kutowutfola lusito emtfolamphilo? (probe: in terms of respectful or disrespectful, kind or unkind, gentle or rough, patient or impatient, etc)?** Please describe or give examples |  |  |  |
|  |  |  |  |
|  |  |  |  |
|  |  |  |  |
|  |  |  |  |
| **Ucabanga kutsi tinjani tinhlelo noma lusito lwetemphilo emangweni wakini?** |  |  |  |
| Very good |  |  |  |
| Good |  |  |  |
| Bad |  |  |  |
| Very bad |  |  |  |
| Comments: |  |  |  |
| **Yini leyakwenta kutsi ukhetse lomtfolamphilo lotsatsa kuwo emaphilisi akho ema-ARVs?** | | | |
| Proximity to home/work | | | |
| Privacy/confidentiality | | | |
| Ease of access | | | |
| Referral from family and friends | | | |
| Trust the clinician | | | |
| Comments | | | |
|  | | | |
| **Nawukhuluma nesisebenti setemphilo (Dokotela/nurse) ngekwelashwa kwakho noma lusito lwetemphilo, yini lebaluleke kakhulu kuwe?** | | | |
| Friendly | | | |
| Respectful | | | |
| Competent | | | |
| Other | | | |
| Comments | | | |
|  | | | |
| **Yini lebaluleke kakhulu kuwe nawelashwa noma utfola lusito lwetemphilo?** | | | |
| Available services | | | |
| Short wait times | | | |
| Other | | | |
| Comments | | | |

*_____________________________________________________________________________________*

***Manje ngicela kukubuta ngendlela lowaphatseka ngayo nawukulolucwaningo lwe-IPT***

**Ngicela ungicocele kutsi yini leyakwenta wangenela lolucwaningo lwe-IPT?.**

Didn’t want to get TB

Pressured by clinician

Thought I’d get special treatment

Comments

**Ngicela ungicocele kutsi kungani ucabanga kutsi bantfu labaphila neligciwane leHIV bavikeliswe nge-IPT?**

HIV gets better

Prevents TB

Live longer

Comments

**Kukhona yini lowamutjela kutsi ukulolucwaningo?**

- No, I did not tell anyone I was in the study

Umakungenjalo, leni?

- Yes, I did tell someone I was in the study.

**Uma akhona, ngubani?** (Can prompt with below choices.)

- Family
- Friends
- Employer
- Others…………………..……………………………………………………………………………………………………………………..

**Uma akhona, Kingani wamtjela?**

**Uma akhona, Ucabanga kutsi kumtjela kwakusita ngandlela-tsite kutsi ucedze emaphilisi akho ekuvikela i-TB? Ngicela uchaze.**

**Kukhona yini lokwahluka endleleni sisebenti setemphilo (nurse/dokotela wakho) labakuchazela ngayo ngemaphilisi ekuvikela i-TB (uma kucatsaniswa nendlela labaye bachaze ngayo emaphilisi ngalokuvamile)?**

- Yes
- No
- I don’t know

Comments

**Ungangichazela kutsi yini lebeyingatfutfukiswa elucwaningweni lolufana nalolu esikhatsini lesitako?**

***Uma umuntfu atsatsa emaphilisi ekuvikela i-TB, kumelwe banatse bawanetse bawacedze emaphilisis abo kute atowubasita ngalokuphelele. Ngifuna kucoca nawe ngekutsi wakhona njani kuwanatsa uwacedze emaphilisis ekuvikela i-TB?***

**Kwakutsatsa sikhatsi lesingakanani kutsi ucedze emaphilisi akho ekuvikela i-TB? ________ months**

**Uma kwakutsatsa ngetulu kwetinyanga letisitfupha, uyakhumbula kutsi kungani kwaba njalo?**

**Yini nje leyakusita kutsi ukhone kutsatsa emaphilisi akho ekuvikela i-TB onkhe-malanga? (Prompt if necessary: kukhona yini lobekukusita kutsi ukhumbule kunatsa emaphilisis akho?)**

**Kwake kwenteka yini kutsi ukhohlwe kunatsa liphilisi lakho nawusebentisa emaphilisis ekuvikela i-TB?**

- No
- Yes

Uma kunjalo, uyakhumbula kutsi kwabangelwa yini loko? (Allow for more than one reason)

- I don’t remember

**Ngicelala ucabange ngelesikhatsi unatsa emaphilisi ekuvikela i-TB.**

**Yini leyakusita kutsi ucedze emaphilisi akho ekuvikela i-TB?**

**Yini nje leyanta kwaba lukhuni/ noma tingcinamba lowabhekana nato lebetibangela kutsi ube nebulukhuni kutsi unatse ecedze emaphilisis akho ekuvikela i-TB?**

**Ucabanga kutsi kuba kulolucwaningo kwakhusita kutsi ucedze emaphilisis akho ekuvikela i-TB? Ngicela uchaze.**

***Kulolucwaningo, bantfu bebabutwa kutsi bebafuna kutwatsatsa kuphi emaphilisi abo – emtfolamphilo noma lapho bahlala khona emamangweni wakubo? Ngifuna kukhuluma nawe ngekukhetsa kwakho?***

**Wajabula yini ngekutsi uvunyewe kutsi ukhetse kutsi ufuna kuwatfola kuphi emmaphilisi ekuvikela i-TB? Leni? (Why or why not)?**

**Ngaletinye tikhatsi, dokotela/nurse wakho uvele akutjele kutsi kufanele uwanatse njani emaphilisi akho ngaphandle wekuva umbono wakho. Ucabangani ngaleyondlela yekukwelapha?**

**Ngaletinye tikhatsi dokotela/nurse akanye nesigulane sakhe bayabonisana ngendlela lengasebenta ekunatseni emaphilisi? Sometimes the patient and clinician make choices about a patient’s treatment together. Ucabangani ngaleyondlela yekukwelapha?**

**Ingabe sisebenti setemphilo (dokotela/nurse) wakutjela/wakukhetsela yini kutsi kumelwe uwatsatse kuphi emaphilisis akho ekuviikela i-TB noma watikhetsela? (Prompt if necessary: Did anyone pressure you to choose getting your pills in the clinic or community?)**

- Yes
- No
- I don’t know

Comments

***________________________________________________________________***

[The next part is **ONLY for those patients who took any of their IPT in the facility-based model i.e., either started or switched to facility-based IPT. If this patient did not take their IPT in a facility, skip this section.]**

***Emarekhodi etfu akhombisa kutsi wakhetsa kutsatsa emaphilisi akho ekuvikela i-TB emtfolamphilo. Ngitakubuta imibuto ngaloko nyalo?***

| **Bowuya ngani emtfolamphilo kuyotfola emaphilisi akho? Check all that apply.** |
| --- |
| Walk |
| Bus |
| Kombi |
| Own Car |
| Other specify |
| Comments |
|  |
| **Ukhona yini lobeketa nawe kulamanye noma cishe onkhe emalanga loweta ngawo emtfolamphilo?** |
| Yes |
| If yes, who? |
| No |

**Ngicela ungicocele kutsi kungani wakhetsa kutfola emaphilisis akho e-IPT emtfolamphilo kunekutsi bakuletsele?**

**Ngicela ungichazele waphatseka njani/wakutfola kunjani kutsatsa emaphilisis akho ekuvikela i-TB emtfolamphilo?**

**__________________________________________________________________________**

**[This part is ONLY for those who took any of their IPT through the community-based model i.e., either started with or switched to community-based IPT. If this patient did not take their IPT in the community, skip this section.]**

***Emarekhodi etfu akhombisa kutsi wakhetsa ktfo lokungenani lamanye emaphilisi akho dvutane nase khaya (emangweni). Ngitakubuta imibuto ngalokukhetsa kwakho nyalo?***

| **Wawuwatsatsa (bebakuletsela) kuphi emaphilisis akho ekuvikela i-TB? Check all that apply.** |
| --- |
| Home |
| Work |
| Church |
| Shop |
| Other specify |
| Comments |
|  |
| **Ukhona yini lobewuba naye kulamanye noma cishe onkhe emalanga ekutfola emaphilisis akho?** |
| Yes |
| Uma kunjalo, bekungubani? |
| No |

**Ngicela ungicocele kutsi kungani wakhetsa kutfola emaphilisi akho e-IPT dvutane emangweni kunekutsi uwalandze emtfolamphilo?**

**Ngicela ungicazele kutsi waphatseka njani / bekunjani nje kutfola emaphilisis akho emangweni. [Possible prompts: Ingabe bekudzingeka ulindze aze nurse afike noma bekafika ngesikhatsi? Ingabe bebabonakala bakajakile? Kukhona mhlawumbe lokunye lokwenteka lokwaba kuhle noma kwangakuphatsi kahle ?**

***Bantfu labangenela lolucwaningo bebaniketwe litfuba lekushintjashintja kutsi bafuna kuwatsatsa kuphi emaphilisi abo e-IPT. Ngifise kububuta kabanti ngaloko.***

**Wake washintja yini lapho bewutsatsa khona emaphilisis akho e-IPT, ngokwesibonelo kushintja ekuwatsaseni ekhaya bese uwatsatsa emtfolamphilo**

- Yes

Uma kunjalo, kungani washintja?

- No, I didn’t switch
- I don’t know

**Wayishintja yini lapho bowutsatsa khona emphilisi akho e-IPT tikhatsi letingetulu kwalesisodwa?**

- Yes

Uma kunjalo, kungani wasintjashintja?

- No, I didn’t switch more than once
- I don’t know

***Njengoba sesicedza, ngingatsandza kwati kutsi ucabangani ngekusebentiswa kwemaphilisi ekuvikela i-TB eveni lonkhe?***

**Ngekubuka kwakho, tiyini tingcinamba letinkhulu letingabangela kutsi bantfu labanengi labadzinga emaphilisi e-IPT bangawatfoli eSwatini?**

**Ngekubuka kwakho, tiyini tingcinamba letinkhulu letingabangela kutsi umuntfu angawacedzi emaphilisi e-IPT?**

**Ucabanga kutsi kubalulekile kutsi emaphilisi e-IPT atsatfwe kanye nema-ARVs?**

- Yes
- No
- I don’t know

Comments

**Ucabanga kutsi kwakusita kutsi ucedze emaphilisi akho kutsi bewuniketwe lilungelo lekutikhetsela kutsi ufuna kuwatsatsa kuphi emaphilisi akho e-IPT? Ngicela uchaze.**

- Yes
- No
- I don’t know

Comments

**Ucabanga kutsi lokwenta kubemelula kuwacedza emaphilisi e-IPT kutsi nurse/ sisebenti selucwaningo besikuphatsa ngendlela lehlukile kulabo labebangakalungeneli lolucwaningo? (Possible prompt: shorter lines, nicer, etc?)**

- Yes
- No
- I don’t know

Comments

***Sibonga kakhulu ngekuba yincenye yalolucwaningo. Nasi sibongo sekuba yincenye yalolucwaningo.***
